# Warm realism in Chinese microdramas: A term frequency analysis of audience comments on *The Puppy Laifu*

**DOI:** 10.1371/journal.pone.0348845

**Published:** 2026-05-11

**Authors:** Yuan Tian, Pan Cheng

**Affiliations:** 1 School of Tourism and Media, Chongqing Jiaotong University, Chongqing, China; 2 Key Laboratory of Complex Systems Optimization and Intelligent Control of Chongqing Municipal Education Commission, Chongqing Jiaotong University, Chongqing, China; 3 School of Mathematics and Statistics, Chongqing Jiaotong University, Chongqing, China; Selcuk University, TÜRKIYE

## Abstract

Starting from the connotation of warm realism, this study analyzes the narrative characteristics of the warm realist microdrama *The Puppy Laifu* on the Douyin platform. The audience’s comments on the drama are selected as the research object. Narratology is also introduced into the short drama for category construction. On the basis of a combination of audience reception theory and word frequency analysis, a “narrative-decoding” analytical framework is constructed to study the narrative setup and audience reception mechanism. The analysis reveals that the emotional resonance of the audience is centered on the active decoding of characters’ morality and competence, a strong critique of the characters’ words and actions, and a scrutiny of the rationality of the plot settings within the story experience. Therefore, narrative designs incorporating moral dilemmas and emotional disparities can significantly increase the willingness to comment. To synergistically amplify overall interaction effectiveness and improve dissemination outcomes, platforms should focus on maximizing audience’ willingness to comment because of the strong positive correlation among four indicators: comments, likes, favorites, and shares. To improve the creation and dissemination of warm realistic microdramas, creators should consider the following suggestions: the use of flat characters to create moral conflict; the establishment of emotional resonance through deliberate emotional contrasts at the plot level; conflict resolution to provide emotional solace; and strengthening of the mechanisms likely to trigger comments.

## 1. Introduction

Because of the increasingly “fragmented and mobile” trends in global digital content consumption, short-form video has evolved into a core media form that transcends cultural and geographical boundaries, becoming a shared focal point in the transformation of the global film and television industry [1]. From the user-generated content systems of YouTube Shorts and TikTok to Netflix’s “Fast Laughs” and Disney + ’s short drama sections, as well as Korea’s Naver TV minidramas and India’s MX Player short-form content, major international platforms have all invested in the short-form narrative space. This shift is not only reshaping narrative models in the global film and television landscape but also driving a cross-cultural convergence in audience viewing habits. Global short-form narrative characteristics have become a universal narrative norm that transcends language and cultural barriers, providing international expression possibilities for local content creation in different countries [2]. In this international landscape, China’s microdramas have developed rapidly, not only providing an important window into digital content innovation but also being exported to many countries and regions around the world through the cross-border platform Reelshort, achieving a leap from local creation to international dissemination.

In 2022, the National Radio and Television of China successively issued the *“Notice on Further Strengthening the Management of Online Micro-Drama and Implementing the Creation Improvement Plan” and the “Opinions on Promoting the Prosperity and Development of Short Drama Creation*”. These policies formally defined microdramas as a distinct genre characterized by brevity and tightly structured narratives. In 2024, the annual releases of traditional TV dramas and online dramas decreased by 22.7% year-on-year, whereas the output of micro-dramas increased by 35%, with the market expected to reach CNY 100 billion by 2027 [3,4]. These trends indicate that microdramas are becoming the core growth pole in China’s film and television industry, and the processes involved in their development have also provided a reference paradigm for short narrative works around the world.

However, with the rapid development of the global short-form narrative industry, some microdramas have also been characterized by narrative homogeneity and ambiguous value orientation, in some cases based on a “popularity-first” attitude [5]. There are some prevailing themes, such as “time-travel” [6], “rebirth” [7] and “a domineering CEO” [8], in China’s microdrama market and in the sensory stimulation targeted by short-form content on international platforms. Some of these microdramas have fallen into the trap of an excessive emphasis on entertainment, while lacking a meaningful portrayal of real life and sincere human relations. In this context, realism has always been an important consideration in the creation of global microdrama narratives; for example, Christopher discussed the queer social realism in London [9], and Rana described the essence of the kitchen sink realism across diverse cultural landscapes [10]. The warm realism paradigm that emerged in Chinese film and television creation is an extension and innovation of global realism creation. Programs grounded in this paradigm achieve the organic unity of aesthetic value and social function through a life-oriented narrative and popular perspective.

The commonality between this paradigm and realism is that both frameworks are committed to depicting the daily life and emotional dilemmas of ordinary people. However, warm realism places greater emphasis on revealing real problems and providing emotional solace and moral hope, which constructs a “criticism-consolation” narrative mechanism. This narrative mechanism retains realism’s focus on social issues while also catering to the audience’s need for emotional resonance. It offers a promising cross-cultural pathway for microdramas to transcend the limitations of mere entertainment and achieve a balance between intellectual depth and communicative appeal. Nevertheless, current academic research on warm realist microdramas focuses predominantly on textual narrative analysis [11,12], with limited attention given to actual audience reception and feedback, making it difficult to fully capture the practical effects of these narratives. Therefore, there is an urgent need to supplement relevant empirical studies from the audience’s perspective.

To empirically examine the narrative characteristics and audience reception mechanisms of warm realist microdramas, this study selects Douyin (China’s TikTok counterpart), the platform with the largest audience in China [13], as the observational field. On the basis of the popularity rankings in December 2024, the warm realist microdrama *The Puppy Laifu* was chosen for research. This drama stands out among microdramas dominated by highly entertaining themes such as “time travel” and “domineering CEOs,” achieving more than 100 million views in a single week.

*The Puppy Laifu* revolves around a left-behind elderly woman and a loyal dog collaborating to rescue a kidnapped child, transforming social governance issues into a narrative practice of moral community reconstruction and reflecting the audience’s preference and value-driven demands for realist microdramas. It is a representative work exploring the narrative mechanisms and audience reception patterns of warm realist microdramas.

Audience comments represent viewers’ interpretations of the text of *The Puppy Laifu*, a process of active participation in meaning construction. In accordance with audience reception theory, audience comments are selected as the core analytical object. On the basis of the audience comments, a “narrative–decoding” analytical framework is constructed. By integrating term frequency statistics and sentiment analysis, this study systematically reveals the audience’s reception tendencies and emotional engagement mechanisms.

In this paper, the characteristics that place *The Puppy Laifu* within the framework of warm realism are delineated from three core dimensions: critical intensity, emotional comfort level, and clarity of solutions. Then, by integrating quantitative tools from classical narratology, audience reception theory, and mathematical linguistics, the narrative essence is analyzed at the story level; the emotional flow and resonance mechanisms are decoded for the audience at the reception level; and the ways in which the narrative settings regulate audience participation is explored from the emotional perspective. This approach addresses how serious social issues can effectively resonate with audiences within a fragmented and entertainment-oriented media environment. Finally, on the basis of the above analysis, actionable narrative strategies are proposed for the creation of warm realist microdramas from four dimensions: character, plot, moral construction, and the enhancement of communication effectiveness. This study not only provides empirical references for the refined creation of microdramas but also offers practical insights into how microdrama narratives can maintain social concern, evoke emotional resonance, and guide audience interactions.

## 2. Narrative theoretical framework

To systematically analyze the narrative mechanism and the pathways to audience resonance in the warm realist microdrama *The Puppy Laifu*, this chapter first defines and integrates the main theories upon which the research is based, and then a narrative categorical framework for analyzing audience comments is constructed.

### 2.1 Warm realism

Warm realism is a narrative paradigm that has emerged and gained widespread attention in the creation and criticism of Chinese film and television in recent years [14]. Its ideological roots can be traced back to the early Chinese film tradition of “educating society,” and it gradually formed a clear aesthetic consciousness through creative evolution after different historical periods. Scholar Hu defines warm realism as follows: “while focusing on ordinary life narratives and confronting real-world dilemmas, warm realism adopts warmth as its dominant tone to express the pursuit of truth, goodness, beauty, brightness, and the future” [15]. This definition accurately portrays the main characteristics of this paradigm: maintaining a dynamic balance among social criticism, emotional comfort, and the clarity of solution.

To prevent the concept of “warmth” from remaining vague in concrete analysis, this study develops three operational analytical indicators based on the three dimensions of criticality, comfort, and solution clarity. First, critical intensity refers to the depth and sharpness of the work’s revelation and reflection on social problems, human dilemmas, or structural contradictions. Second, emotional comfort denotes the level of emotional support and psychological solace provided to the audience during the narrative process. Finally, the clarity of the solution refers to the specificity and credibility of the paths to conflict resolution presented within the drama.

Building upon this foundation, this study proposes a more inclusive and dynamic definition of warm realism: warm realism is a creative paradigm that systematically adjusts the relationships among critical intensity, emotional comfort, and solution clarity within a narrative form. It does not shy away from profound revelations of real-world contradictions, instead engaging in medium-to-high critical intensity. Simultaneously, it strives to provide continuous emotional support and psychological consolation throughout the narrative process, providing medium-to-high levels of emotional comfort. Furthermore, the narrative guides the audience through a meaningful transformation from revealing problems to instilling hope through symbolic or emotional solutions, presenting high-clarity solutions.

*The Puppy Laifu* exemplifies a synergistic configuration across these three dimensions: it possesses a medium-to-high level of critical intensity, directly addressing social issues such as left-behind elderly and child safety; it provides medium-to-high emotional comfort by soothing internal conflicts and dangers through the emotional symbol of Laifu, thereby constructing positive emotional experiences; and it provides high solution clarity, endowing the narrative with consolation and hope through clear moral appeals. Therefore, this drama is a typical warm realist microdrama, not only in its pursuit of emotional authenticity but also in its comforting nature. It not only presents dilemmas but also strives to showcase the possibilities and directions for overcoming them.

### 2.2 Story and discourse

Classical narratology, as shown by Amerian and Jofi, focuses on the internal structural logic of texts and the dynamic interplay among narrative components [16]. Events in stories are always represented in a certain mode, whether textual, cinematic, choreographic, or visual, to mediate between the *story* and *discourse* [17]. To make the application of narratology to microdramas more organized, the concepts are described below.

Guided by classical narratological principles [18], this study examines the narrative architecture of *The Puppy Laifu* with a story-level focus, deliberately excluding discourse analysis (e.g., narrative temporality or perspective shifts). This methodological delimitation stems from the empirical reality that 99.7% of audience comments engage exclusively with story elements while showing negligible interest in narrative devices.

Here, “story” serves as the analytical nucleus and is defined as a chronological sequence of causally linked events, encompassing plot dynamics, character arcs, and environmental contexts. Because microdramas prioritize visceral storytelling over stylistic experimentation, the structural components of plot, character, and environment are the primary carriers of audience engagement. This framework decodes how warm realism transmutes social critique into emotionally resonant narratives.

Aristotle defined plot as the arrangement of the incidents in Poetics [19]. After Aristotle, traditional conceptions of plot tended to treat it as the content of stories, emphasizing the arrangement of incidents. In contrast, classical narratology focuses on relationships between narrative elements, derives plot structures such as narrative grammar and chronological order, and entirely disregards character psychology and reader response [20]. To address the limitations of classical narratology, postclassical narratology emerged and addresses the interactions between narrative and external factors (readers, culture, media) [21]. Following postclassical narratology, because the comments from the audience pertaining to the plot are analyzed in the present study, ‘story experience’ is incorporated as a plot dimension.

In narratology, scholars have established two primary frameworks for analyzing characters: the psychological perspective, which examines internal traits such as motivations and emotions, and the functional perspective, which focuses on roles and actions within narrative structures [22]. In the context of microdrama audience comments, discussions predominantly focus on characters’ moral qualities and personality traits. Even when viewers reference characters’ actions, their critiques or praises ultimately aim to highlight underlying qualities. Consequently, this study prioritizes the psychological perspective in its categorical framework, as it aligns with audience preoccupations and the analytical focus on character-driven emotional resonance.

Environmental elements have long been integral to literary story structures, serving as foundational components of narrative design [23]. When the structures of microdramas are analyzed, the environmental elements are reconceptualized as narrative space, a term that involves locations and environments. This narrative space primarily integrates the visual characteristics of cinematic storytelling within the “story” domain and incorporates lens space at the physical level [24]. Thus, the narrative space discussed in the microdrama is divided into two components: lens space and geospatial space. Geospatial space refers to the background settings and geographical environments, whereas lens space encompasses spatial relationships in frame composition, as well as diverse spatial constructs created through audio‒visual combinations and shot compositions.

Many researchers pay attention to the application of narration in microdramas. Hanney suggested that microdrama is a form of data-driven storytelling from a narratological perspective and that the deployment of cliffhangers is important [4]. Considering the platform and the characteristics of microdramas, Li proposed that narrative objects should adapt to changes [5]. Most studies have focused on the text itself. Here, a categorical framework for story analysis is established on the basis of audience comments, as shown in Table 1.

**Table 1:** Categorical framework.

Category	Variable Coding
Plot	1: Plot Discussion; 2: Story Experience; 3: Others
Characters	1: Appearance & Physique; 2: Morality & Competence; 3: Acting Performance; 4: Others
Narrative Space	1: Geospatial Space; 2: Lens Space (Frame Composition, Audio-Visual Combinations, Special Effects, etc.); 3: Others

### 2.3 Audience reception theory

As an “encoded text,” the microdrama *The Puppy Laifu* embodies a clear encoding of dominant ideology through its fast-paced, high-conflict, and value-driven narrative design. Viewers’ interpretations of its authenticity, values, and solutions reveal significant room for negotiation, which aligns closely with Stuart Hall’s “encoding/decoding” model and the subsequent audience reception theories [25].

The core idea of this theory lies in the fact that the meaning of a text is not transmitted unidirectionally but rather actively constructed by the audience during the reception process [26]. On the basis of their own experiences and social positions, audiences may adopt a dominant, negotiating, or adversarial interpretation. Therefore, comments are not merely feedback but also a visible arena for viewers to negotiate the meaning of the text, express emotions, and engage in social interaction.

By combining classical narratology with audience reception theory, an integrated analytical framework is formed: *The Puppy Laifu* encodes specific moral conflicts and emotional nodes within its narrative, which is decoded by evoking audience emotions and judgments and ultimately manifests as quantifiable interactive behaviors such as comments and likes. This framework systematically explains how narrative elements, such as characters and plot, become the core mechanisms driving audience engagement.

### 2.4 Term frequency analysis

Term frequency analysis (TFA) of audience comments in microdrama represents an interdisciplinary fusion of linguistics and screen media studies [27]. The application of mathematical methods to analyze linguistic patterns originated in literary text analysis—exemplified by T.C. Mendenhall’s 1887 statistical study of Shakespearean works [28]. With the turn of the 20th century, mathematical linguistics emerged as a new discipline. The application of this method has achieved notable progress, enabling breakthroughs in syntactic analysis, information processing, and linguistic quantification [29].

Within microdrama research, high-frequency term extraction remains a cornerstone methodology for dissecting audience feedback [30]. The process begins by identifying statistically prominent terms within a corpus, as their frequency often correlates with representational salience. A higher term frequency typically indicates that the term is more representative within the specific text [31]. By conducting term frequency analysis on microdrama comment texts, it is possible to effectively identify words with frequencies significantly higher than the benchmark value, thereby intuitively revealing the core dimensions of audience focus and their emotional orientations.

In summary, on the basis of the integration of term frequency analysis and narratological category construction, this paper conducts empirical research on microdrama comments following the steps of text collection, data cleaning, word frequency statistics, and interpretation of the results.

## 3. Text collection

### 3.1 Case selection

Given the sheer volume of daily microdrama releases on Douyin, this study focuses on warm realist microdramas that premiered on Douyin during 2024. Three criteria are used for this selection: The microdrama must be a warm realist work released in 2024; it must have entered the top 10 of Douyin’s microdrama popularity rankings on the second day after its release; and it must have garnered a substantial number of viewer comments. Ultimately, *The Puppy Laifu* was selected as the case among warm realist microdramas that simultaneously fulfill the requirements of timeliness, viewership, and comment volume because of the more than 100 million views and over 1,500 comments within two weeks of its release, reaching sixth place on the popularity chart on December 7th, the day after its premiere.

In a rural context, the drama focuses on conflicts such as urban–rural divides, attitudes toward pets, and child trafficking. The characters are distinctly layered in the drama. The grandmother, Yao Yao, and Laifu form the core positive characters, and the human traffickers are the typical antagonists, while the son and daughter-in-law function as morally flawed protagonists with a bias against rural life and aversion to the pet. The couple’s stubbornness and cognitive limitations escalate the narrative tension, reflecting the value conflicts in real society and reinforcing the realistic characteristics of the drama.

### 3.2 Text scraping and data cleaning

*The Puppy Laifu* comprises 30 episodes, structured across three tiers of audience access. Episodes 1–10 are fully open to all viewers with unrestricted commenting privileges. Episodes 11–12 were temporarily available without payment during their promotional window, with comment functionality enabled only during this period. Episodes 13–30 require either a paid subscription or viewing a 15-second advertisement for free access, and commenting on these episodes is restricted to paid subscribers. As of February 6, 2025, there were 6,893 comments on the first 10 episodes and 1,328 comments on the subsequent 20 episodes. A total of 8,221 audience comments were collected manually from all 30 episodes. Each comment was treated as an independent textual unit for analysis.

Given the highly unstructured and noisy nature of audience comments in microdramas, rigorous text cleaning was implemented to isolate meaningful and contextually relevant data. The 8,221 raw text units underwent systematic proofreading to remove advertisements, emojis, unrelated remarks, comments lacking logical coherence and entries with fewer than two characters. Following this process, 4,095 valid comments were retained.

This study employs the coding classification strategy in Table 1 and uses Weiciyun software to segment the corpus into words. A word frequency table is generated by sorting the words in descending order of frequency, and representative high-frequency words are selected for in-depth analysis.

## 4. Term frequency statistical analysis

### 4.1 Comment text classification and analysis

Given that most audience comments on microdramas generally exhibit characteristics of ultrashort texts, traditional clustering algorithms such as TF-IDF are difficult and inadequate for calculating semantic relevance and categorizing such textual data [32]. On the basis of the categorical framework in Table 1, this study adopted a manual annotation strategy for classification conducted by two authors.

To ensure the accuracy and objectivity of the manual classification results, the coding of comments was carried out jointly by two authors after the data were cleaned. Prior to the coding process, the two coders had in-depth discussions to fully understand the categorical framework and coding rules. Five hundred comments were randomly selected from the 4,095 valid comments and coded independently on the basis of the classification framework established in Table 1. After coding, a reliability test was conducted using Cohen’s kappa coefficient. The coefficient for the plot category was 0.85, and the coefficient for the character category was 0.81, both greater than 0.8, meeting the reliability requirements. The first author subsequently completed the classification of the remaining comments. The classification results are presented in Table 2.

**Table 2:** Categorical frequency statistics (N = 4095).

Category	Options	Frequency	Percentage	Total
Plot	1: Plot Discussion	627	15.3%	46.7%
	2: Story Experience	1003	24.5%	
	3: Others	282	6.9%	
**Characters**	1: Appearance & Attire	25	0.6%	53.2%
	2: Morality & Abilities	2011	49.1%	
	3: Acting Performance	90	2.2%	
	4: Others	53	1.3%	
**Narrative Space**	1: Geospatial Space	2	0.05%	0.1%
	2: Lens Space	2	0.05%	
**Total**		4095	100%	100%

The comments on *The Puppy Laifu* are predominantly focused on characters and plot, with little attention given to narrative space, such as cinematography, audiovisual composition, or color design. The two most commented-on categories are “Characters 2” (morality and abilities) and “Plot 2” (story experience), which reflect the audience’s primary concerns. Most of the comments (2,011 entries, 49.1%) are on “Character 2”.The audience focused intensely on whether characters were portrayed as kind, morally upright, and capable in their actions. Discussions on “Plot 2” accounted for 24.5% of the comments. These categories reflect audience engagement with narrative logic and emotional resonance and include debates over plot plausibility or reactions to character-driven conflicts. The narrative space element accounted for only 0.1% of the comments, indicating that the drama met basic standards in scene construction and audiovisual language, without any obvious audiovisual embellishments or production flaws that would spark discussion.

As shown in Table 2, character-related comments dominated audience engagement, constituting the highest proportion of feedback. Viewers directly praised or criticized characters through remarks such as “This family is even worse than animals” and “The old lady’s son is a beast,” underscoring their intense focus on moral oppositions and value conflicts within the narrative. Plot-related discussions accounted for 46.7% of the comments, reflecting deep audience investment in the storyline. Examples of critiques include, “The telescope lens was not open,” “Shouldn’t someone have called the police immediately? What a brainless drama!” and “Instead of going out to search, they just wail at home”, exemplifying viewers’ meticulous scrutiny of plot plausibility and logical consistency. When identifying narrative loopholes, audiences actively debated these issues with others, fostering communal discussions about the drama’s plausibility.

In contrast, elements such as acting skills, physical appearance, and costume design received minimal attention. Unlike “domineering CEO” dramas, which prioritize glamorous aesthetics, comments on appearance and attire constituted less than 1% of the total feedback. This aligns with the principles of warm realism, where character designs eschew exaggerated distinctions between beauty and ugliness, instead mirroring ordinary individuals to enhance relatability.

While the classification of comments confirms audiences’ strong interest in characters and plot, the analysis of high-frequency terms is necessary to pinpoint the specific aspects of character development (e.g., moral integrity, competence) and plot elements (e.g., tension points, resolution strategies) that most effectively capture viewer attention.

### 4.2 Statistical analysis of high-frequency terms

High-frequency terms, as indicators of audience consensus, directly reflect the core narrative elements of the microdrama. Their statistical representation reflects both the distribution patterns of the focuses of audience perception and the visible changes in the text’s narrative characteristics. The degree of repetition of specific terms directly reflects the intensity of group resonance associated with the narrative elements they represent.

Audience comments on plot and characters carry different emotional tendencies. This study employs a bipolar sentiment analysis of audience comments: positive and negative emotional tendencies. This emotional distribution pattern mirrors the dual narrative mechanism of “critique–consolation” in warm realism. It both promotes critical thinking through value conflicts and achieves emotional comfort by promoting justice. To conduct the term frequency analysis, the two researchers analyzed and synthesized the sample comments and extracted separate corpora for characters, plot, story experience, and sentiment. These corpora were integrated with Weiciyun software to generate term frequencies for each corpus type, and the following analysis was conducted.

#### 4.2.1 High-frequency terms on characters.

The character dimension accounts for 53.2% of all the comments, with corresponding high-frequency sentiment terms exhibiting distinct emotional polarity. Evaluations surrounding core characters show significant moral polarization. Among character-level comments, terms related to the dog are mentioned most frequently. First, the frequencies of various nicknames for the dog Laifu were counted, as shown in Table 3. Then, using Weiciyun software, the co-occurrence frequency of each sentiment term with these nicknames was generated. Finally, the emotional terms about the dog were selected and are listed in Table 3 with their corresponding frequencies. The sum of the co-occurrence frequencies of each sentiment term with all the nicknames for the dog were also calculated using simple addition and are listed in Table 3.

**Table 3:** Co-occurrence statistics of sentiment and Puppy-related terms.

**Character Nicknames**	**Term**	**Laifu**	**Puppy**	**This Dog**	**Dahuang**	**Little Dog**	
	**Frequency**	440	396	152	96	92	
**Sentiment Orientation**	**Term**	**Smart**	**Great**	**Human-like**	**Cute**	**Loyal**	**Liked**
	**Frequency**	450	407	123	389	95	91
**Co-occurrence Frequency of Sentiment and Characters**		438	400	105	381	88	87

Among the 4,095 valid comments, approximately one-third centered on Laifu (the puppy), with its nicknames emerging as the most frequently mentioned term. Co-occurrence analysis revealed overwhelmingly positive evaluations of Laifu, dominated by descriptors such as “smart,” “cute,” and “loyal.” This finding fully aligns with the creators’ intention to portray the puppy as a “moral exemplar of loyalty, bravery, and selflessness,” with the audience unreservedly embracing this emotional symbol.

The second most frequently mentioned terms in character-level comments refer to the child’s parents—the elderly woman’s son and daughter-in-law (hereinafter referred to as “the couple”). Similar to the results presented in Table 3, emotional terms expressing the couples’ emotional inclinations are listed in Table 4 with their frequencies. The sentiment terms and their corresponding frequencies are listed separately, and the sum of the co-occurrence frequencies between each sentiment word and the various nicknames for the couple is calculated through simple addition. The results are presented in Table 4.

**Table 4:** Co-occurrence statistics of sentiment and couple-related terms.

**Character Nicknames**	**Term**	**Woman**	**Son**	**Couple**	**parents**	**Spouses**	**Daughter-in-law**	**Man**
	**Frequency**	352	352	295	180	153	104	98
**Sentiment Orientation**	**Term**	**Inferior to**	**Stupid**	**Furious**	**Clumsy**	**Idiotic**	**Pig**	**Deserve It**
	**Frequency**	568	390	176	156	97	96	78
**Co-occurrence Frequency of Sentiment and Characters**		566	382	167	145	89	90	73

Co-occurrence frequency analysis between sentiment-oriented terms and nicknames for the couple indicates that audience evaluation lies in the negation of their cognitive abilities and behavioral efficacy. Among these, the highest-frequency sentiment terms include “inferior to”, “worse than animals,” “worse than pigs,” “worse than beasts,” and similar expressions. The couple is depicted as flat characters with substantial limitations in capability and obvious cognitive flaws, often portrayed as well-intentioned but ultimately causing harm. Audience emotional fluctuations are primarily directed at this cognitively deficient couple. Such strong moral outrage stems precisely from the audience’s identification with value standards such as kindness and competence, representing a natural reaction to positive characters who deviate from these standards. This reaction is the result of dominant decoding.

In summary, the drama strategically focuses on the internal moral conflict within the positive camp in its character setting. This powerful focus is achieved by creating a sharp contrast between the virtuous, intelligent, and capable Laifu and the couple with cognitive deficiencies. The drama uses realistic social elements such as mother-in-law/daughter-in-law conflicts, urban‒rural divides, and differing attitudes toward pet ownership to shape these intentionally unlikable flat characters. Aligning with the principle of warm realism, which focuses on ordinary individuals, positive characters are not portrayed as dazzling or heroically righteous, while the antagonist (the human trafficker) is not depicted as utterly evil or grotesque and instead appears as an ordinary person. This setup directs the audience’s attention away from traditional good-versus-evil oppositions and toward the conflicts arising from internal contradictions within the positive faction. Such conflicts touch upon universally shared values among the audience, compelling viewers to shift from passive watching to active judgment. Consequently, the comment data, both in quantity and tendency, reveal a high concentration of affirmation toward positive characters, negation of cognitively deficient characters, and a notable disregard for the antagonist.

#### 4.2.2 High-frequency terms pertaining to plot.

Audience commentary on the plot predominantly centered on questioning narrative plausibility and articulating emotional reactions to key story developments. The corresponding high-frequency terms and their frequencies are systematically categorized and presented in Table 5:

**Table 5:** High-frequency terms in plot setup and story experience.

**Plot Setup**	**Term**	Call the police	Bite	Rescue	Location tracking	Open		
	**Frequency**	155	102	90	75	36		
**Story Experience**	**Term**	Angry	Beat	Scold	Disbelief	Cannot do	Look down on	Ignore
	**Frequency**	143	122	96	84	74	53	38

The audience exhibits particular sensitivity in scrutinizing plot logic, often commenting on elements in the drama that defy common sense or have logical weaknesses. On the basis of discussions about “plot design,” typical plot loopholes include positive characters who forget to call the police when faced with dilemmas and delayed medical treatment for the injured dog in the finale (episode 30). Viewers do not deny the story’s ultimate value and goal, but on the basis of their own life experience and common sense, they raise localized critiques and optimization suggestions related to the “clarity of solutions”. This negotiated decoding approach demonstrates the deep engagement of audiences, who desire the story to be more self-consistent within a logic framework they can accept. Although such narrative designs can effectively stimulate discussion and increase popularity, they often evoke negative emotions such as dissatisfaction or even disappointment among viewers. Therefore, such loopholes should not be overly frequent or conspicuous, as they may lead audiences to perceive the work as poorly crafted or lacking rigor, thereby negatively impacting the overall viewing experience.

The plot design downplays the traditional good-versus-evil opposition, instead emphasizing internal conflicts within the righteous camp. This makes the audience’s regret over the good guys’ mistakes and the story’s deviation from its ideal direction particularly prominent in their “story experience” comments. As a result, in story experience, the audience’s profound distress over the mistakes of the protagonists and their lament over the story’s deviation from an ideal direction become particularly prominent. This is especially evident in discussions about the behavior of the couple in the drama, with comments such as “These two are driving me crazy” and “I really want to punch this man and woman”. The reason lies in the audience’s complete internalization of the moral standards and values established by the script, leading to righteous condemnation of characters who deviate from these values. This dominant decoding indicates that the audience has deeply immersed itself in the moral order preconstructed by the story and actively assumes the role of a “moral adjudicator.”

In summary, viewers primarily engage in dominant decoding in terms of character and story experience, while they engage in negotiated decoding in plot settings. Dominant decoding is often accompanied by strongly emotional and forceful expressions in the drama. This demonstrates the resonance between viewers’ emotional inclinations and the set emotion in the microdrama—namely, a genuine affirmation of integrity and kindness and a sharp critique of human flaws. As Syrdal demonstrated, content that evokes high-arousal emotions is most effective at driving user engagement [33]. This strong and widespread emotional response needs to be expressed. On instant interactive platforms, posting comments becomes the most direct channel for venting emotions and seeking validation. This precisely highlights the essence of warm realism—achieving emotional solace and affirmation of the light of humanity through viewers’ shared indignation and anticipation.

## 5. Communication effects and narrative design

On short video platforms, metrics such as likes, comments, favorites, and shares serve as pivotal quantitative indicators for measuring the dissemination effectiveness of microdramas, directly reflecting audience engagement and approval [34]. Optimizing these engagement metrics is therefore critical for enhancing dissemination outcomes. This study examines the dynamic interplay and interactive mechanisms among these four metrics within audience comments on The Puppy Laifu, aiming to extract audience-centric narrative elements. The findings provide actionable strategies for genre-specific microdramas to amplify communication efficacy through intentional narrative engineering.

**Fig 1:**
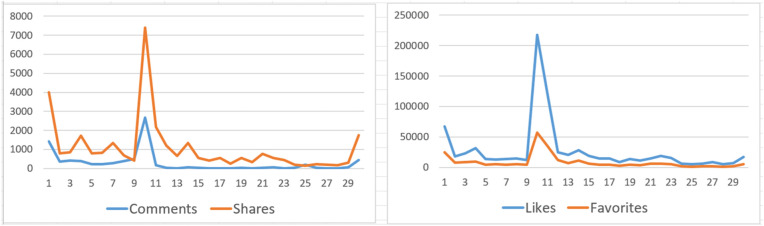
Data of comments, shares, likes, and favorites.

As of February 6, 2025, the data collected on the four factors of likes, comments, favorites, and shares in *The Puppy Laifu* are listed in Fig 1. The correlation coefficients calculated using the Pearson correlation coefficient formula are shown in Table 6.

**Table 6:** The correlation coefficients of metrics.

	Likes	Comments	Favorites	Shares
**Likes**	1			
**Comments**	0.832	1		
**Favorites**	0.989	0.827	1	
**Shares**	0.922	0.941	0.931	1

As illustrated in Table 6, the correlation coefficients among the four engagement metrics of comments, likes, favorites and shares all surpass 0.8, demonstrating their highly interconnected dynamics within *The Puppy Laifu*. This robust interdependence suggests that amplifying one metric (e.g., comments) is likely to catalyze proportional increases in others [35]. Upon deeper analysis, likes, favorites, and shares, considered only as quantitative data, carry relatively surface-level information about audience feedback. In contrast, comments uniquely encapsulate rich qualitative insights into audience psychology, behavioral patterns, and perceptual engagement [36]. The content of comments not only includes individuals’ immediate reactions to the work but also fosters collective emotional resonance and linguistic interaction. Therefore, deeply stimulating comment interactions and encouraging audience expression are likely to significantly increase the effectiveness of series dissemination.

In *The Puppy Laifu*, the distribution of comments across the final 18 episodes (paid comment episodes) is relatively stable, generally ranging between 5 and 79 per episode. However, the comment counts for Episodes 25 and 30 increased to 195 and 456, respectively, showing significant peaks. This phenomenon highlights the strong stimulating effect of the specific narrative design in these two episodes on audience willingness to comment. Therefore, in this study, the valid comments of these two episodes are encoded with high-frequency word analysis to investigate the reasons behind the surge in audience engagement.

The narrative of Episode 25 focuses on the couple’s repeated blunders that endanger Laifu’s life, intensifying narrative stakes. The comments reveal that 71.4% of the feedback focused on “Characters 2: Morality & Abilities”, indicating that audience emotional engagement was driven primarily by the portrayal of the couple’s flaws. High-frequency term analysis underscores that the couple was mentioned 98 times, with 96 instances co-occurring with negative sentiment terms such as “stupid” and “clumsy”. This overwhelming disapproval reflects the audience’s rejection of the couple’s moral hypocrisy and cognitive ineptitude, particularly their failure to protect the protagonist Laifu. Through the erroneous actions of the internal characters, the audience’s strong disapproval and moral critique of the couple intensified to a peak, maximizing viewer engagement and emotional investment. Concurrently, questioning the rationality of the couple’s cognitive flaws also constitutes the behavioral logic and psychological motivation behind the audience’s negotiated decoding.

Episode 30 portrays Laifu’s tragic death during the rescue of the child from human traffickers, marking the narrative’s emotional apex. Postcoding analysis revealed that 46.7% of the comments fell under “Plot 2: Story Experience”, overwhelmingly reflecting the audience’s psychological responses to the climax. High-frequency term extraction identified 250 emotional words (e.g., “heartbreaking,” “devastated”), vividly encapsulating viewers’ profound sorrow and collective grief. The audience was profoundly moved by Laifu’s selfless sacrifice and unwavering loyalty, which served as pivotal emotional development. The audience’s emotions and values were in perfect alignment with the work’s final thematic direction. Through the hero’s sacrifice and the satisfying conclusion of the child’s rescue, the anger and frustration from previous episodes were transformed into a deeper, more profound sense of being moved and moral identification.

The above analysis reveals that *The Puppy Laifu* guides viewers to actively decode the story and activates their willingness to comment and interact through two key narrative designs. On the one hand, it deliberately created internal conflicts of morality and abilities at the character level; on the other hand, it designed “suffering” scenes with emotional disparities. Viewers engaged in high-intensity critiques of certain social realities within a dominant framework. Subsequently, a consolatory ending with high emotional comfort consolidated these emotions, transforming them into dominant value identification and emotional resonance. The audience’s experience was not just one of despair but also included warmth and hope with clear solutions, thereby accomplishing effective social emotional guidance and mainstream value dissemination. Consequently, in the narrative strategy of the microdramas, systematically designing key narrative points can precisely trigger audience emotional resonance and ultimately reinforce emotional engagement and value identification. Such designs not only effectively enhance the willingness to comment but also synergistically amplify overall interaction effects according to the strong positive correlations among metrics such as comments, likes, favorites, and shares, thereby improving dissemination effectiveness.

## 6. Recommendations for optimizing the narrative

This study conducts term frequency analysis within a narratological framework to systematically quantify and reveal that the core driving force of audience interaction originates from moral dimensions and emotional contrasts at the narrative level. Drawing on Hall’s decoding theory, it is evident that audiences engage in re-creation of the text through both dominant decoding based on value identification and negotiated decoding based on logical plausibility, thereby validating the high consensus potential and emotional mobilizing capacity of warm realist themes. To clarify the optimal narrative path for warm realist microdramas, this paper proposes the following recommendations from the perspectives of character, plot, meaning construction, and dissemination drivers.

First, the character construction emphasizes the narrative leverage and decoding hub role of flat characters. *The Puppy Laifu* fully aligns with the creative principles of warm realism by rejecting visual spectacle and the opposition of physical beauty versus ugliness. Its characteristics embody an everyday aesthetic that remains close to reality. The portrayal of flat characters focuses on depicting their singular yet prominent cognitive flaws. The conflicts in words and actions arising from these cognitive flaws lead the protagonist into perilous situations, becoming a strong decoding focus. Within the dominant decoding framework, audiences reaffirm and defend the value standards preset by the script, with 49.1% of the comments focusing on the characters’ traits and abilities. Therefore, at the narrative character level, emphasis should be placed on portraying characters’ universally relevant moral dilemmas and cognitive limitations, making them a common subject for the audience’s moral judgment and emotional projection. It is advisable to design functional flat characters that can advance the plot and bear critical intensity as narrative levers, thereby effectively stimulating the willingness to comment and consequently enhancing dissemination effectiveness.

Second, the plot deliberately creates emotional resonance after emotional disparities to trigger strong emotional fluctuations in the audience’s story experience. The cognitive deficiencies of flat characters cause the story to deviate from the audience’s expected trajectory and harm the positive characters, evoking strong emotional resonance in the audience. This also stimulates their willingness to comment and vent their emotions through real-time comments. When members of the audience express their story experience, they make strong moral judgments about the controversial behavior of flat characters. This also prompts the audience to examine the rationality of plot development, thereby naturally transitioning to negotiated decoding. Such story experiences, which trigger emotional resonance, represent a narrative strategy that guides audiences from passive reception to active participation. Therefore, designing emotional resonance after emotional disparities in the plot not only enhances the audience’s narrative involvement and cognitive participation but also creates enormous emotional tension. This deep triggering of empathy and moral indignation thus stimulates commenting behavior.

Furthermore, shared values are established to achieve emotional solace. *The Puppy Laifu* downplays the traditional binary opposition, instead intensifying internal conflicts within the righteous camp. By avoiding scenes of gore and violence, it avoids creating an atmosphere of oppression and despair. While presenting dilemmas, it ultimately guides the narrative toward moral perseverance and hope through issues of high moral consensus, comfort with high emotional solace, and solutions with high clarity. This is one of the distinctive features of warm realist microdramas. Therefore, it is essential not only to construct an emotional loop of “critique-consolation” but also to provide clear, credible, and positive emotional compensation and value-driven resolution in the creative process. This approach facilitates the audience’s transition from emotional catharsis to value identification at the audience level, making dominant decoding the mainstream and incorporating negotiated decoding as an organic component of interactive engagement.

Finally, the comment trigger mechanism should be strengthened to synergistically enhance the dissemination effect. This arises from the close correlation among the four metrics of dissemination effectiveness. The comment peaks observed in episodes 25 and 30 demonstrate the substantial leveraging effect of specific narrative settings on interactive behavior. Narrative nodes capable of arousing emotions can be directly translated into driving forces for comments. Since comments serve as the core linking likes, favorites, and shares, during the narrative design phase, it is essential to consciously plan decoding-trigger scenarios with strong emotional resonance. This synergistically enhances the overall dissemination effect and influence of the work.

In summary, the narrative foundation should be anchored in genuine social concerns, maintaining a medium to high level of social critique to establish the story’s credibility and value for discussion. Emotional design must incorporate elements of medium-to-high emotional comfort while embedding conflict that can elicit the audience’s emotional judgment. The creative loop needs to provide a high-definition solution, guiding the audience from emotional catharsis to emotional sublimation through value recognition. The dissemination strategy should emphasize the driving role of comments, embedding high-arousal emotional content at key narrative nodes to effectively incentivize interaction, thereby enhancing the dissemination effectiveness and social impact of the work. Only by achieving a balance among artistic expression, emotional resonance, and social responsibility can realist microdramas truly forge a high-quality development path that is both warm and profound, critically acclaimed and commercially successful.

## 7. Limitations and future research

As a representative case of a warm realist microdrama, *The Puppy Laifu* offers important insights into narrative strategies and dissemination effects. However, to more objectively achieve universal relevance in the creation and dissemination of warm realism microdramas, it is necessary to rationally examine the specificities of the sample used in this study and the scope of applicability of its conclusions.

Several limitations of this study must be acknowledged. The first is the specificity of the narrative medium. While the drama employs an animal hero as an emotional bond and moral embodiment, this nonhuman emotional medium, although highly impactful, cannot be applied to all social issues. In most cases, warm realism must confront the complexities of human nature and the struggle for reconciliation. Its key lies in constructing a pure emotional connection that transcends language and speaks directly to the heart. The second is self-selection bias in the data. This study relies on publicly available comments voluntarily posted by viewers. Active commenters are often users with strong emotions and a high willingness to express themselves, who may not fully represent the entire audience. This bias may be further amplified for the latter 20 episodes, where commenting was restricted to paying users, tilting the sample toward the core audience. The conclusions drawn are more likely to reflect the reception patterns of highly engaged viewers. The third is the dual high cost of narrative and audience reception. Warm realism works often require that creators have the abilities to balance social insight, logical truth, and emotional depth while also requiring greater emotional investment and cognitive load from the audience. Therefore, within a microdrama market driven by “instant gratification” and algorithm-based recommendations, this genre must remain a high-quality, slow-burning form, struggling to overcome the high-cost issue.

Future research can be further deepened and expanded in the following directions. First, a bias-correction mechanism can be introduced to optimize the research design. To avoid self-selection bias, a hybrid research method combining comment analysis, such as questionnaire surveys and in-depth interviews, can be adopted. The bias in the comment data can be corrected and supplemented, improving the reliability of the research conclusions. Second, the emotional semantics of emoticons may be preserved and analyzed with a refined sentiment analysis system. During the comment-cleaning process of this study, emojis were removed, which may have led to the loss of rich emotional information and reduced the accuracy of the sentiment analysis. Future research could introduce an emoticon sentiment dictionary and semantic mapping model to accurately decode the audience’s emotional tendencies and intensity, deepening the understanding of cross-cultural emotional resonance mechanisms. Finally, the scope of the research sample can be expanded to increase the generalizability of the conclusions. In this study, only a single microdrama was used as the analysis sample. The uniqueness of the sample may limit the generalizability of the conclusions. Future research could select warm realist microdramas from different regions, themes, and creative styles as study samples, making the conclusions more universally applicable and practically instructive.

## Supporting information

S1 Data Dataset Fig 1. (XLSX)
